# Scrapie Control in EU Goat Population: Has the Last Gap Been Overcome?

**DOI:** 10.3389/fvets.2020.581969

**Published:** 2020-09-29

**Authors:** Sergio Migliore, Roberto Puleio, Guido Ruggero Loria

**Affiliations:** Istituto Zooprofilattico Sperimentale Della Sicilia “A. Mirri”, Palermo, Italy

**Keywords:** TSEs: transmissible spongiform encephalopathies, scrapie, PRNP: prion gene name, goat, EU regulation, genetic—disease resistance, biodiversity

## Introduction

Scrapie is a fatal, neurodegenerative disease that affects sheep and goat worldwide, belonging to the group of transmissible spongiform encephalopathies (TSEs).

Since 2002, Member States (MS) of European Union (EU) have implemented active surveillance to control the risk of scrapie. The EU scrapie eradication policy is mainly aimed to eradicate classical scrapie. The choice of population groups and sample sizes have evolved in the years, as well as the eradication measures and control of disease (selective culling, movement restrictions, reinforced surveillance measures, etc.). In this context, over the past two decades, breeding programs to increase the frequency of the resistance-associated ARR allele in sheep populations have been introduced to minimize TSE risk in MS, but there was not a regulatory effort in adoption of analogous measures for goats. However, scientific knowledge related to scrapie resistance associated with goat PRNP gene polymorphisms has considerably expanded in the last 10 years.

Classical scrapie is considered endemic in many MS. Since its publication, the only measures applicable for TSE control in goat contained in Regulation (EC) No 999/2001 obliged farmers to provide a complete culling of whole flock, with great economic loss and serious concerns for the risk of extinction of endangered breeds. However, over the years, additional measures have been introduced such as monitoring of the infected herd without the obligation of total culling and the possibility of reintroducing goats with unknown genotype after biosafety practices. Nevertheless, these measures could allow the goat population to become the main reservoir of scrapie, affecting the disease eradication program in small ruminant population.

Following a request from the European Commission (EC), the European Food Safety Authority (EFSA) was asked to deliver scientific opinions on the scrapie situation in EU to evaluate the introduction of breeding policies in goats. From 2014, EFSA advised to promote selection and introduction of resistant bucks in EU caprine population (1). More recently, in 2017, based on the latest scientific evidence, EFSA concluded that breeding programs for scrapie resistance in goats should be implemented in MS, taking particular attention to potential negative effects of extinction in rare and endangered breeds (2).

With Regulation (EC) No 2020/772 of June 11, 2020, amending Regulation (EC) No 999/2001, EC laid down new approaches as regards eradication measures for TSEs in goats and in endangered breeds. In this context, the authors discuss advantages and critical points related to the different control measures introduced by EU regulations during the last two decades.

## State of the Art

### Legislative Basis

Regulation (EC) No 999/2001 establishes rules for the prevention, control, and eradication of certain TSEs, including scrapie in small ruminants. This Regulation dates back to 2001 and, after many subsequent amendments, is still in force today.

In 2003, Regulation (EC) No 260/2003 revised the requirements for eradication measures in case of the detection of TSE in a farm by selective culling of susceptible sheep and by requiring the implementation of measures to increase TSE resistance in the outbreak. Simultaneously, decision 2003/100 (EC) laid down requirements for the establishment of breeding programs for resistance to TSE in sheep, aimed to increase the level of alleles associated with resistance (ARR) and decreasing the frequency of alleles associated with susceptibility (VRQ) in EU sheep population. Commission Regulation (EC) No 1923/20065 and No 727/2007 then integrated the breeding program requirements into Regulation (EC) No 999/2001. In 2006, EFSA confirmed the efficacy of breeding program for TSE resistance in sheep (3).

More recently, on June 11, 2020, Regulation (EC) No 2020/772 amended Annexes I, VII, and VIII to Regulation (EC) No 999/2001 introducing the possibility for the MS to limit slaughtering/culling and destruction to goats which are genetically susceptible to classical scrapie. In addition, the definition of “endangered breed” of Regulation (EU) 2016/1012 replaced the expression of “local breed in danger of being lost to farming” as laid down in Regulation (EU) No 807/2014 (4).

### Scrapie in EU Goats

Classical scrapie shows similar epidemiological features in sheep and goats and the involvement of both species in outbreaks is common. Even if the incidence in goats is much lower than in sheep, milk and placenta of infected goats may serve as sources of infection to sheep (5, 6). Scrapie in goat was described for the first time in 1942 (4); since then, clinical cases have been recorded throughout Europe. Animal movements between herds and environmental contamination play relevant roles as risk factors.

In 2019, a total of 325,386 sheep and 138,128 goats were tested in EU. In sheep, 821 cases of classical scrapie were detected in seven MS, whereas 517 cases were reported in goats in seven MS (7). Scrapie in goat is considered endemic in the EU countries with the largest caprine populations with more than 10,500 cases from 2002 to 2017. Between 2002 and 2015, classical scrapie was detected in 10 MS with 2.4 cases out of 10,000 tested heads. In this prevalence study, Cyprus was excluded due to an epidemic over the last 10 years (2).

### Genetic Basis

In the last two decades, an extensive review of literature was conducted to identify relevant alleles of goat PRNP to which a breeding program could be based. These studies were conducted within different MS and goat breeds. A considerable dataset has been produced for the following alleles: S127, M142, R143, D145, D146, S146, H154, Q211, and K222. Among them, K222, D146, and S146 alleles confer higher genetic resistance to classical scrapie strains circulating in the EU goat population (2). In 2017, based on a combination of the “weight of evidence” and the “strength of resistance,” EFSA provided a ranking of resistance to classical scrapie, as follows: K222 > D146 = S146 > Q211 = H154 = M142 > S127 = H143 > wild type (2).

### Goat Breeding in EU

Goat farming plays an important socioeconomic role in several countries, particularly where there are hills and mountains, and remote, marginal, and even semi-arid areas (8, 9). Europe is the continent with the widest caprine biodiversity with 187 goat breeds, which is 33% of the goat breeds acknowledged worldwide (10). In this context, there are breeds with large population sizes, cosmopolitan and often characterized by a high production, and breeds with small population sizes not yet subjected to conservation programs because of their remoteness or because they are less competitive in terms of production than other selected breeds (9). Such different scenarios obviously have required a different scrapie control strategy.

## Discussion

In 2017, EFSA, based on prolonged field experience and experimental studies, concluded that the K222, D146, and S146 variants confer genetic resistance to the classical scrapie strain circulating in the EU goat population (2). EFSA highlighted that the protective effect of K222 is greater than D146 and S146 variants and of ARR allele in sheep, when the 2002 Scientific Steering Committee opinion was published (2). In this regard, a substantial difference between sheep and goats in the new Regulation (EC) No 2020/772 still remains. In sheep, the ARR/ARR homozygous genotype in reproductive males is an essential requirement, whereas in goats, heterozygosity for at least one of K222 and D/S146 alleles is sufficient to avoid the stamping out. It should be remembered that heterozygous variants Q222K and N146S/D in goats do not confer full protection against classical scrapie as reported in natural outbreaks in Greece (11) and in Cyprus (12). In addition, the subsequent restocking of outbreak without genotype consideration after biosafety practices is a considerable risk. These are critical points whose efficacy will be assessed in the future.

The EFSA opinion also highlights that a high selective pressure in some breeds with a low frequency of resistant variants would likely have an adverse effect on genetic diversity and that each MS should be able to design its own genetic selection strategy depending on the breed concerned.

Estimating the frequency of candidate alleles is a preliminary step in understanding the feasibility of a breeding program. Several investigations on goat PRNP were performed in MS in recent years, and some breed-related differences emerged (Table 1). Higher frequency (>24.5%) of 146D or S variants was described in cosmopolitan Boer goat in Great Britain and Netherlands (13–15) and in native Damascus and related breeds in Cyprus (16.5%) (17). A lower frequency (3%) was also described in local and crossbred in Greece (16). To date, this mutation does not seem to be widespread in other MS. In contrast, 222 K variant seems to be more common across the MS. Frequencies between 1.2 and 7.5% were described in cosmopolitan and large population size breeds such as Saanen (1.2–4%) and Alpine (6.4–7.5%) reared in Spain, Netherlands, Italy, France, and Greece (15, 16, 18–20). Very high frequency (29.5%) was described in Dutch Toggenburg in Netherlands (15). Variable frequencies were described in small size of native breeds such as local and crossbred in Greece (0.3–5.6%) (16). In Italy, where a great caprine biodiversity is present, a difference between northern and southern native breeds was described (20), with higher frequencies of 222 K in Southern breed such as Garganica (17.2%), Ionica (7.2%), southern crossbred (22.5%), Girgentana (18.7%), Rossa Mediterranea (12.7%), Argentata dell'Etna (16.3%), Aspromontana (10.3%), and Cilentana (18.2%) (20–23). Many of these breeds are considered in critical or endangered status (24) and for this reason any breeding program should consider the endangered status of each goat population to preserve the genetic variability and the biodiversity together with disease control (21).

**Table 1:** Breeds with S146/D146 and K222 haplotypes reported in literature and their frequencies reported in EU.

**Country**	**Breed**	**Geographical classification**	Local status^*^	**Frequency (%)**	**Source**
**146D or 146S**					
UK	Boer	Cosmopolitan	At risk	24.5–35.5	(13, 14)
Netherlands	Boer	Cosmopolitan	Unknown	31	(15)
	Nubian	Cosmopolitan	At risk	7.1	
Greece	Local/crossbred	Local	Unknown	3.0	(16)
Cyprus	Damascus and related breeds	Cosmopolitan	Not at risk	16.5	(17)
**222K**					
UK	Toggenburg	Cosmopolitan	At risk	1.9	(13)
Netherlands	Saanen	Cosmopolitan	Unknown	1.9	(15)
	Toggenburg	Cosmopolitan	At risk	29.5	
Greece	Local/crossbred	Local	Unknown	5.6	(16)
France	Saanen	Cosmopolitan	Not at risk	4.9	(18)
Spain	Saanen	Cosmopolitan	Unknown	1.2	(19)
	Alpine	Cosmopolitan	Unknown	6.4	
	Local breeds	Local	At risk	0–0.03	
Italy (Northern breeds)	Camosciata	Cosmopolitan	Not at risk	2.4	(20)
	Saanen	Cosmopolitan	Not at risk	3.0	
	Roccaverano	Local	Endangered	4.3	
	Valdostana	Local	Critical	1.3	
Italy (Southern breeds)	Garganica	Local	Endangered	17.2	
	Jonica	Local	Critical	7.3	
	Southern crossbred	Local	Unknown	22.5	
	Girgentana	Local	Endangered	18.7	(21)
	Rossa Mediterranea	Local	Critical	12.7	(22)
	Argentata dell'Etna	Local	Endangered	16.3	
	Aspromontana	Local	No at risk	10.3	(23)
	Cilentana	Local	Critical	18.2	

* *DAD-IS database—FAO (22)*.

Various mutations in the PRNP in different breeds have potentially been positively selected in relation to local circulating scrapie strains originating in specific environmental conditions (25).

A recent study (26) assessed the impact of different breeding strategies in goat using a mathematical model, and it concluded that breeding programs for scrapie resistance could be implemented also in a context of so high biodiversity and also different size of the populations of goats. Nevertheless, the growth rate of resistant goats in some breeds may be slow due to the initial genetic profile not being particularly favorable inside the breed. In cosmopolitan breeds with a large population size, a breeding program in the overall population would be desirable. In contrast, in endangered breeds with a small population, a breeding program should be implemented starting from reproductive nuclei. This scheme is less expansive and protects the endangered breeds even if it takes longer to reach the expected results.

As well as goat breeds, a breeding program for scrapie resistance should consider the particular situation of each MS in terms of the presence of resistant alleles and their relative frequency. For example, in Greece, which has one of the largest goat populations in Europe, a goat-scrapie resistance program targeting the Q211, S146, and K222 alleles was designed (27), whereas in Italy, pilot projects selected positively a singular variant K222.

Although there is a strong interest in disease control among goat farmers in the Northern MS, breeding for resistance is often compromised by the low frequency of resistant alleles. By contrast, in Southern MS where a satisfying frequency of resistant alleles is present, goat farming is mainly related to pastoralism and in several cases there is a lack of interest in starting genetic programs. For this reason, to be successful, new regulations have to consider engaging farmers' cooperation by appropriate risk communication and involving them in the genetic program as well as providing an adequate financial support for goat genotyping.

Regulation (EC) No 2020/772 laid down an alternative tool for scrapie control in EU goat population. It particularly recognized the genetic resistance to classical scrapie in goats carrying at least one of the most recognized alleles (K222, D146, and S146) and preserving them from culling in the case of outbreak. In addition, the new regulation introduces possible derogation measures for endangered breeds according to Regulation (EU) 2016/1012. This new measure will finally strengthen the control of TSEs in small ruminants in the EU and will also have beneficial effects on farming system and for the conservation of goat breed biodiversity.

## Author Contributions

SM drafting of the article. RP and GL revised the article. All authors contributed to the article and approved the submitted version.

## Conflict of Interest

The authors declare that the research was conducted in the absence of any commercial or financial relationships that could be construed as a potential conflict of interest.
